# Can leadership quality buffer the association between emotionally demanding work and risk of long-term sickness absence?

**DOI:** 10.1093/eurpub/ckab090

**Published:** 2021-07-05

**Authors:** Reiner Rugulies, Jeppe Karl Sørensen, Ida E H Madsen, Mads Nordentoft, Kathrine Sørensen, Elisabeth Framke

**Affiliations:** 1 National Research Centre for the Working Environment, Copenhagen, Denmark; 2 Department of Public Health, University of Copenhagen, Copenhagen, Denmark; 3 Department of Psychology, University of Copenhagen, Copenhagen, Denmark; 4 Institute for Molecular Medicine Finland, University of Helsinki, Helsinki, Finland

## Abstract

We examined whether the association between emotionally demanding work and risk of register-based long-term sickness absence (LTSA, ≥6 weeks) was buffered by high leadership quality among 25 416 Danish employees during 52-week follow-up. Emotional demands were measured at the job group level, whereas leadership quality was measured by workers rating their closest manager. Emotionally demanding work was associated with a higher risk of LTSA, regardless if leadership quality was high or low, with neither multiplicative nor additive interaction. We conclude that we found no evidence for high leadership quality buffering the effect of emotionally demanding work on risk of LTSA.

## Introduction

Emotionally demanding work requires sustained emotional effort by the worker, for example dealing with sorrows or aggressions by clients, patients or customers.^1^ Studies have reported associations between emotional demands and risk of long-term sickness absence (LTSA), although it is debated whether these associations indicate causality.^2–4^

We recently reported prospective associations between emotionally demanding work and LTSA in the Work Environment and Health in Denmark (WEHD) study.^4^ Working in an emotionally demanding job predicted risk of LTSA with a hazard ratio of 1.32 [95% confidence interval (CI): 1.14–1.52].^4^

Considering the consistent association between emotional demands and LTSA in the literature^2–4^ and considering that high emotional demands are a part of work in numerous occupations, for example, health care and teaching,^4^ we aimed to examine whether other, more modifiable, work environment factors could act as buffers. We chose leadership quality, reasoning that workers may be able to cope with emotional demands if their managers are competent, caring and supportive.^5^ Previous studies reported inconsistent associations between leadership quality and LTSA;^6^ however, a recent analysis of WEHD data showed that high leadership quality predicted a reduced risk of LTSA.^6^ To our knowledge, no study has examined yet whether leadership quality can buffer the effect of emotionally demanding work on risk of LTSA.

We therefore re-analyzed our recently published data on emotional demands and LTSA^4^ and examined whether this association was modified by worker-rated exposure to high or low leadership quality.

## Methods

### Study design and participants

WEHD is a biennial survey in a nationwide sample of employees residing in Denmark aged 18–64 years.^4^ We combined data from the 2014 and 2016 waves, comprising 33 544 respondents (response rate: 49.4%), representing a wide range of jobs in the Danish workforce. We excluded respondents who were out of the labour market, with previous LTSA or missing values, yielding a sample of 25 416 workers [13 576 women (53.4%), mean age 45.1 years (standard deviation: 11.4)].^4^

### Emotional demands

Participants responded to two questions about perceived emotional demands (frequency of being emotionally affected by work) and content-related emotional demands (frequency of contact with individuals in difficult situations) at work, respectively. We aggregated scores on each item to the job group level using the International Standard Classification of Occupations and assigned these job group means to all individuals of the same job group.^4^ We categorized individuals as working in high emotionally demanding jobs if their job title scored above the mean on both perceived and content-related demands (Supplementary material 1).

### Leadership quality

Leadership quality was measured with an eight-item scale, filled-in by the workers, covering collaborative, supportive, caring and appreciative behaviours of their managers. The items loaded on one global factor and the scale had a high internal consistency.^6^ Using median split, we distinguished between low and high leadership quality (Supplementary material 2).

### LTSA

Using the Danish Register for Evaluation of Marginalisation (DREAM),^7^ we identified onset of LTSA during 1-year follow-up. In line with previous research,^4^^,^^6^ we included sickness absence spells lasting ≥6 weeks, the minimum time for registration in DREAM.^4^ We only included first-time LTSA spells, as these differ from recurrent spells.^8^

### Statistical analysis

Using logistic regression analyses, we calculated odds ratios (OR) and 95% confidence intervals (CI), testing the effect of an interaction between emotional demands and leadership quality on risk of LTSA during 52 weeks of follow-up. Estimates were adjusted for age, sex, education, cohabitation, children living at home and year of survey. Departure from multiplicativity was analyzed by inserting the product of the dichotomized emotional demand and leadership quality variables into the logistic regression analysis. Departure from additivity was analyzed by calculating the relative excess risk due to interaction (RERI), the attributable proportion due to interaction (AP) and the synergy index (S). Confidence intervals were calculated with the Hosmer and Lemeshow method.^9^ We estimated number of cases by multiplying cases per 1000 person-years in the reference group with the ORs.

To assess a possible effect of low statistical power we conducted supplementary analyses by adding 25 759 responders from the 2012 wave of WEHD (where emotional demands were measured slightly differently) and re-ran the analysis with 51 175 responders.

The study was approved by The Danish Data Protection Agency (no. 2015-57-0074). In Denmark, studies based on questionnaire and register-data only do not require approval from the National Committee on Health Research Ethics.

## Results

During 52 weeks of follow-up, 1061 participants (4.2%) became LTSA cases, including 625 (3.6%) and 436 (5.4%) with low and high emotionally demanding jobs, respectively.

High emotionally demanding work predicted risk of LTSA regardless if leadership quality was high (OR: 1.39, 95% CI: 1.11–1.73) or low (OR: 1.39, 95% CI: 1.15–1.68) (table 1). The OR for interaction as departure from multiplicativity was 1.01 (95% CI: 0.95–1.08).

**Table 1:** Effect modification by leadership quality of the association between working in an emotionally demanding job and risk of long-term sickness absence in 25 416 respondents from the Danish workforce

	Exposed	Cases		Departure from multiplicativity		Departure from additivity		Estimated number of cases per 1000 person-years	
	*N*	*N*	%	OR	95% CI	OR	95% CI	*N*	95% CI
Workers reporting high leadership quality									
Low emotionally demanding jobs	8801	258	2.9	Reference		Reference		30	27 to 34
High emotionally demanding jobs	3921	180	4.6	1.39	1.11 to 1.73	1.42	1.16 to 1.75	43	38 to 48
Workers reporting low leadership quality									
Low emotionally demanding jobs	8600	367	4.3	Reference		1.45	1.23 to 1.70	44	39 to 49
High emotionally demanding jobs	4094	256	6.3	1.39	1.15 to 1.68	1.97	1.63 to 2.38	60	53 to 67
Odds ratio (OR) for the interaction term of emotionally demanding work times leadership quality (95% CI)				1.01 (0.95 to 1.08)					
Relative excess risk due to interaction (RERI) (95% CI)						0.10 (−0.28 to 0.48)			
Attributable proportion due to interaction (AP) (95% CI)						0.05 (−0.14 to 0.24)			
Synergy index (S) (95% CI)						1.12 (0.73 to 1.71)			

Adjusted for age, sex, education, cohabitation, children living at home and year of survey.

Using the combination of low emotionally demanding jobs and reporting high leadership quality as the reference group, ORs were 1.42, 1.45 and 1.97 for high demands/high leadership quality, low demands/low leadership quality and high demands/low leadership quality, respectively. Neither RERI, AP nor S indicated interaction as departure from additivity.

Supplementary analyses with a larger sample yielded similar results (Supplementary material 3).

## Discussion

We found no evidence that high leadership quality buffers the association between emotionally demanding work and risk of LTSA. We had previously reported that both emotionally demanding work^4^ and low leadership quality^6^ predicted LTSA, and, unsurprisingly, participants exposed to both factors had the highest risk of LTSA in the present study. However, interaction analyses did not indicate effect modification.

### Comparison with previous studies

To our knowledge, no previous studies had examined a buffering effect of leadership quality on the association between emotional demands and sickness absence. Madsen et al. previously showed that leadership quality did not modify the association between emotional demands and antidepressant treatment.^5^ Török et al. reported that a high level of workplace social capital, which may conceptually overlap with leadership quality, did not buffer the associations between workplace violence and LTSA.^10^ Thus, our study add to a growing evidence that workplace resources, such as high leadership quality and high workplace social capital, are associated with a lower risk of sickness absence^6^^,^^10^ but that they do not buffer the effect of adverse working conditions, including emotionally demanding work.^5^

### Limitations

We did not have information on diagnoses and therefore could not differentiate between sickness absences due to physical versus mental health problems. Data were available for sickness absence of ≥6 weeks only, and results may have been different for shorter periods. We did not have information on managers’ demographic or job characteristics that may have modified results. We could not examine the role of leadership quality in jobs of extremely high emotional demands, as this would have required a larger sample size. As LTSA may be affected by national legislations, replications of this study in national workforces of other countries are recommended.

## Conclusion

We found no evidence that high leadership quality may buffer the association between emotionally demanding work and risk of LTSA. Thus, focussing on improving leadership quality seems unlikely to mitigate potentially adverse health effects of emotionally demanding work. There is a need for examining other work environment factors than leadership quality that may buffer potentially adverse effects of emotionally demanding work.

## Funding

This study was funded by the Danish Work Environment Research Fund (grant numbers 27-2017-03 and 10-2016-03). The funding source had no role in the following: study design; the collection, analyses and interpretation of data; the writing of the manuscript; or the decision to submit the manuscript for publication.

*Conflicts of interest*: None declared.

## Ethics approval

The study was approved by The Danish Data Protection Agency through the joint notification of the National Research Centre for the Working Environment (no. 2015-57-0074). In Denmark, studies that are based on questionnaire and register-data only do not require approval from the National Committee on Health Research Ethics.

## Data availability

Data may be obtained from a third party and are not publicly available. Data are stored in a protected server environment hosted by Statistics Denmark. Under certain conditions, qualifying researchers may get access to the data. Please contact the first author, Professor Reiner Rugulies (rer@nfa.dk), for details.

**Key points**

- Emotionally demanding work is associated with risk of poor health and long-term sickness absence.
- As emotionally demanding work is part of the job in numerous occupations involving person-related work, there is a need to identify workplace factors that may buffer its potentially adverse effects.
- High leadership quality by the workers’ closest manager did not buffer the association between emotionally demanding work and risk of long-term sickness absence in the Danish workforce.
- The results suggest that a focus on improving leadership quality at work is unlikely to mitigate the potentially adverse health effects of emotionally demanding work.
- There is a need for examining other factors than leadership quality that may buffer the potentially adverse effects of emotionally demanding work

## Supplementary Material

ckab090_Supplementary_Data
