# Supplementary material for: Can leadership quality buffer the association between emotionally demanding work and risk of long-term sickness absence?
Source: Eur J Public Health. 2021 Jul 5;31(4):739–41. doi: 10.1093/eurpub/ckab090 (PMC8561255; doi:10.1093/eurpub/ckab090)
Supplement: ckab090_Supplementary_Data [file ckab090_Supplementary_Data.docx]

**Supplementary material to article:**

**Can high leadership quality buffer the association between emotionally demanding work and risk of long-term sickness absence?**

Rugulies R, Sørensen JK, Madsen IEH, Nordentoft M, Sørensen K, Framke E.

- Supplementary material 1: Measuring emotional demanding work
- Supplementary material 2: Measuring leadership quality
- Supplementary material 3: Re-analysis with a larger study sample

**Supplementary material 1: Measuring emotional demanding work**

**A) Items**

**Perceived emotional demands at work**

- “How often are you emotionally affected by your work?”

**Content-related emotional demands at work**

- “How often at work are you in contact with individuals in difficult situations (individuals who for example, are affected by serious disease, accident, grief, crisis, or social problems)?’.

Response categories: 1: Never; 2: Seldom; 3: Sometimes; 4: Often; 5: Always.

**B) Calculating the dichotomous variable “emotional demanding work” at the job group level**

The methods for calculating the dichotomous variable “emotional demanding work” is described in detail in an article by Framke et al. 2019 published in Occupational and Environmental Medicine. Below, we briefly summarize the method:

- We calculated the mean scores for “perceived emotional demands at work” and for “content-related emotional demands at work” for each job group defined by the three-digit level of DISCO-08, the Danish version of the International Standard Classification of Occupations (ISCO)-08 system. There were 138 different job groups included in the sample.
- We assigned these mean scores to all individuals of the same job group.
- We plotted the job group mean scores of perceived and content-related emotional demands against each other to visually display each job (see Figure 2 in Framke et al. 2019). Using the scatter plot, we identified job groups scoring above the mean on both perceived and content-related emotional demands.
- The group means across all job groups were 2.8 (standard deviation: 1.0) and 3.0 (standard deviation: 1.2) for perceived and content-related emotional demands, respectively.

Reference:

Framke E, Sørensen JK, Nordentoft M, Johnsen NF, Garde AH, Pedersen J, Madsen IEH, Rugulies R. Perceived and content-related emotional demands at work and risk of long-term sickness absence in the Danish workforce: a cohort study of 26 410 Danish employees. Occupational and Environmental Medicine. 2019;76(12):895-900. <http://dx.doi.org/10.1136/oemed-2019-106015>

**Supplementary material 2: Measuring leadership quality**

**A) Items**

- How often does your immediate manager explain the company’s objectives so you understand what they mean for your work tasks?
- How often do you have sufficient authority in relation to your responsibilities at work?
- How often does your immediate manager take the time to engage in your professional development?
- How often does your immediate manager involve you in the planning of your work?
- How often does your immediate manager give you the necessary feedback (praise and criticism) for your work?
- How often is your work recognized and appreciated by the management?
- How often do you get the necessary help and support from your immediate manager?
- How often can you trust the information that comes from the management?

Response categories: 1: Never; 2: Seldom; 3: Sometimes; 4: Often; 5: Always.

**B) Calculating the dichotomous variable “leadership quality” at the individual level**

- We calculated the mean scores for “leadership quality” for each participant by summing up the scores for each of the 8 items, resulting in a score ranging from 8 to 40.
- The mean score was 27.4 with a standard deviation of 6.6.
- We dichotomized leadership quality in low and high by median split. The median was 28.

For more information on the psychometric properties of the leadership quality scale see the article by Sørensen et al. 2020 in Journal of Occupational and Environmental Medicine.

Reference:

Sørensen JK, Framke E, Clausen T, Garde AH, Johnsen NF, Kristiansen J, Madsen IEH, Nordentoft M, Rugulies R. Leadership quality and risk of long-term sickness absence among 53,157 employees of the Danish workforce. Journal of Occupational and Environmental Medicine. 2020;62(8):557-565. <http://dx.doi.org/10.1097/JOM.0000000000001879>

**Supplementary material 3: Re-analysis with a larger study sample**

**Table S1. Effect modification by leadership quality of the association between working in an emotionally demanding job and risk of
long-term sickness absence in 51,175 respondents from the Danish workforce (WEHD 2012, 2014 and 2016)**

|  | **Exposed** |  | **Cases** | |  | **Departure from multiplicativity** | |  | **Departure from additivity** | |  | **Estimated number of cases per 1,000 person-years** | |
| --- | --- | --- | --- | --- | --- | --- | --- | --- | --- | --- | --- | --- | --- |
|  | **N** |  | **N** | **%** |  | **OR** | **95% CI** |  | **OR** | **95% CI** |  | **N** | **95% CI** |
| **Workers reporting high leadership quality** |  |  |  |  |  |  |  |  |  |  |  |  |  |
| Low emotionally  demanding jobs | 17,308 |  | 544 | 3.1 |  | Reference |  |  | Reference |  |  | 32 | 30 to 36 |
| High emotionally  demanding jobs | 7,888 |  | 373 | 4.7 |  | 1.32 | 1.14 to 1.54 |  | 1.36 | 1.18 to 1.57 |  | 44 | 38 to 50 |
|  |  |  |  |  |  |  |  |  |  |  |  |  |  |
| **Workers reporting low leadership quality** |  |  |  |  |  |  |  |  |  |  |  |  |  |
| Low emotionally  demanding jobs | 17,844 |  | 782 | 4.4 |  | Reference |  |  | 1.38 | 1.23 to 1.54 |  | 44 | 39 to 49 |
| High emotionally  demanding jobs | 8,135 |  | 493 | 6.1 |  | 1.32 | 1.15 to 1.51 |  | 1.78 | 1.55 to 2.03 |  | 57 | 50 to 65 |
|  |  |  |  |  |  |  |  |  |  |  |  |  |  |
| **Odds ratio (OR) for the interaction term of emotionally demanding work times leadership quality (95% CI):** | | | | | | 1.01 (0.97 to 1.06) | |  |  |  |  |  |  |
|  | | | | | |  |  |  |  | |  |  |  |
| **Relative excess risk due to interaction (RERI) (95% CI):** | | | | | |  |  |  | 0.04 (-0.21 to 0.29) | |  |  |  |
| **Attributable proportion due to interaction (AP) (95% CI):** | | | | | |  |  |  | 0.02 (-0.12 to 0.16) | |  |  |  |
| **Synergy index (S) (95% CI):** | | | | | |  |  |  | 1.06 (0.75 to 1.48) | |  |  |  |

Adjusted for age, sex, education, cohabitation, children living at home, year of survey
